# IGFBP7 and Heart Failure: From Senescence Biomarker to Therapeutic Target

**DOI:** 10.1007/s11897-026-00750-3

**Published:** 2026-04-21

**Authors:** Maissa El-Qendouci, Raluca Chelu, Mahmoud Abdellatif, Pardeep Jhund, Rudolf A. de Boer, Navin Suthahar

**Affiliations:** 1 Department of Cardiology, Thorax Center, Cardiovascular Research Institute, Erasmus MC, P.O. Box 2040, 3000 CA Rotterdam, The Netherlands; 2 Department of Cardiology, Medical University of Graz, Graz, Austria; 3 BHF Glasgow Cardiovascular Research Centre, School of Cardiovascular and Metabolic Health, University of Glasgow, Glasgow, United Kingdom

**Keywords:** IGFBP7, Cellular senescence, Heart failure, Biomarker, Therapeutic target

## Abstract

**Purpose of Review:**

To integrate clinical and preclinical evidence on insulin-like growth factor-binding protein-7 (IGFBP7) in heart failure (HF) and identify key priorities for advancing IGFBP7-targeted therapies toward human translation.

**Recent Findings:**

Circulating IGFBP7 is strongly associated with HF development, diastolic dysfunction, and disease progression across HF phenotypes. Preclinical studies show that genetic or pharmacologic inhibition of IGFBP7 attenuates cardiac remodelling, reduces cardiomyocyte senescence, and ameliorates cardiac dysfunction in murine HF models. Apparent mechanistic discrepancies between studies likely reflect cell-specific actions: cardiomyocyte-derived IGFBP7 activates IGF-1 receptor signalling, promoting cardiomyocyte hypertrophy and senescence, whereas endothelial-derived IGFBP7 inhibits cardiomyocyte insulin receptor signalling, impairing metabolic homeostasis.

**Summary:**

IGFBP7 is a senescence-associated biomarker linked to HF development, diastolic dysfunction, and HF progression. Preclinical studies converge on the conclusion that IGFBP7 inhibition attenuates cardiac remodelling and ameliorates cardiac dysfunction in experimental HF, positioning IGFBP7 as a promising therapeutic target. Ongoing research into IGFBP7-targeted strategies may expand HF treatment beyond conventional hemodynamic and neurohormonal interventions by directly addressing the biology of cardiovascular ageing and senescence-driven myocardial dysfunction.

**Graphical Abstract:**

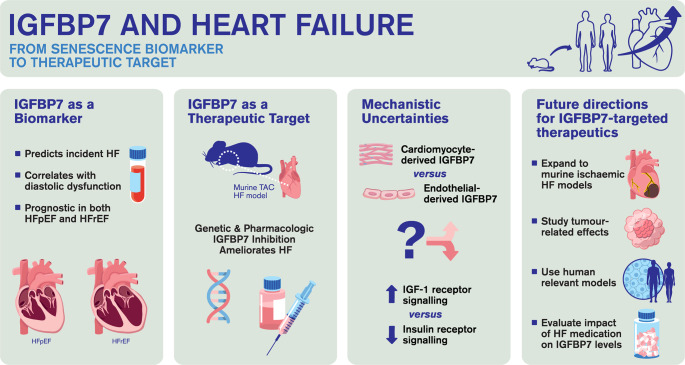

## Introduction

Heart failure (HF) is a complex clinical syndrome characterized by progressive cardiac remodelling and declining myocardial function [1]. Despite major advances in pharmacological and device-based therapies, survival remains poor, with approximately half of HF patients dying within five years of diagnosis [2, 3]. Current therapies predominantly target hemodynamic stress and neurohormonal activation [4, 5]; however, accumulating evidence implicates inflammageing as an additional driver of HF, with senescence-related pathways increasingly recognised as modifiable contributors to disease development and progression [6, 7].

This evolving understanding is grounded in advances in geroscience [8], a field focused on the biological mechanisms of ageing as modifiable determinants of chronic disease. These insights have reframed cardiovascular ageing from a passive, inevitable process to an active, modifiable contributor to disease - in which senescence of diverse cardiac cell types (characterised by permanent cell-cycle arrest and associated functional and inflammatory changes) directly promotes myocardial stress and dysfunction [9]. This conceptual shift has accelerated interest in senotherapeutics, which aim to selectively eliminate or modulate senescent cells, as well as broader gerotherapeutic strategies targeting multiple ageing pathways - collectively offering promising avenues for disease-modifying interventions in HF [10].

The insulin-like growth factor (IGF) signalling axis is a central regulator of ageing across species and organ systems, including the heart [11], partly through modulation of cellular senescence [12, 13]. Among senescence-associated mediators linked to this pathway, insulin-like growth factor-binding protein-7 (IGFBP7) has emerged as a compelling molecular link between ageing biology and clinical HF. A translational study by Chugh et al. in 2013 first implicated IGFBP7 in HF; in a transgenic mouse model of severe cardiac hypertrophy and HF, 52 proteins were differentially expressed in failing hearts, with IGFBP7 showing the largest (35-fold) increase [14]. Subsequent clinical studies confirmed elevated plasma IGFBP7 levels in HF patients compared to controls, supporting its role as a circulating biomarker [14]. Since these pioneering findings, IGFBP7 has consistently demonstrated prognostic value across the HF spectrum [15–17]. More recently, mechanistic studies in murine HF models showed that genetic or pharmacologic inhibition of IGFBP7 attenuates adverse cardiac remodelling and ameliorates cardiac dysfunction – indicating a contributory role of IGFBP7 in HF pathophysiology [18, 19].

Although IGFBP7 is increasingly recognised as both a biomarker and a mechanistic mediator in HF, a focused review on this topic is currently lacking. This review integrates clinical and preclinical studies on IGFBP7 in HF and identifies key priorities for advancing IGFBP7-targeted therapies toward human translation.

## Circulating IGFBP7: Origin and Measurement

**Fig. 1:**
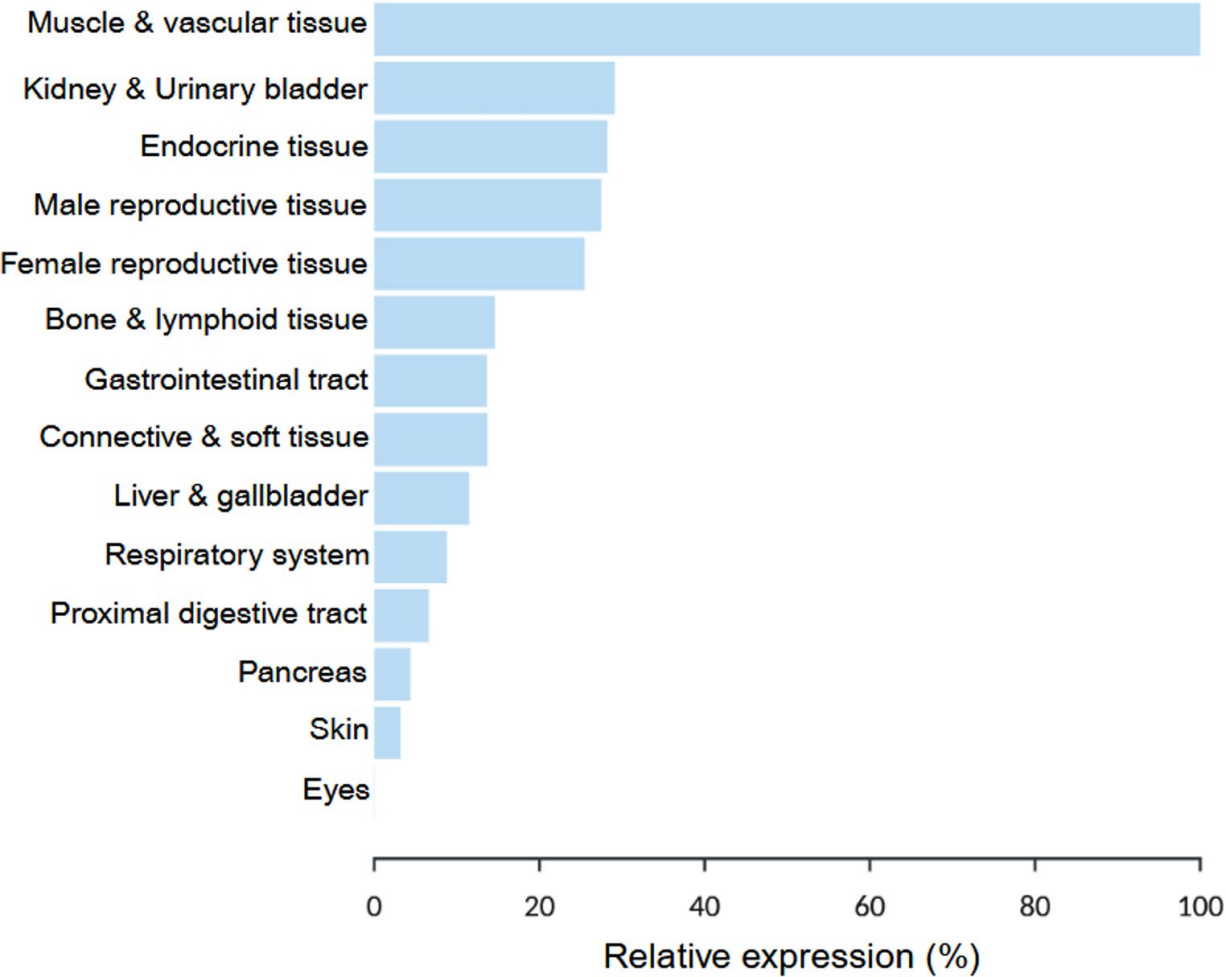
Relative expression of IGFBP7 across various human tissues. Relative expression across tissues was calculated using data from the Human Protein Atlas accessed on 18 February 2026 [20]

In clinical studies, IGFBP7 concentrations are typically measured in serum or plasma and occasionally in urine. IGFBP7 is broadly expressed across multiple organs (Fig. 1) [20], with the highest RNA expression detected in vascular tissue, muscle tissue (cardiac muscle expression > skeletal and smooth muscle expression), and renal tissue, suggesting that these organs may contribute substantially to the circulating pool in healthy individuals. The primary sources of circulating IGFBP7 in human HF, however, remain to be elucidated. Importantly, IGFBP7 is not cardiac-specific, and its expression in other tissues may confound the interpretation of circulating levels in HF patients. For instance, IGFBP7, in combination with urinary tissue inhibitor of metalloprotease-2 (TIMP-2), has been validated as a urinary biomarker of early acute kidney injury, highlighting its systemic regulation beyond the heart [21–24].

Table 1 summarizes the major assay platforms available to measure circulating IGFBP7. The majority of studies used the Elecsys^®^ electrochemiluminescence immunoassay (Roche Diagnostics), which combines high sensitivity (lower detection limit of 0.01 ng/mL) with excellent precision (intra- and inter-assay coefficients of variation of 2–5%). Other platforms, including standard enzyme-linked immunosorbent assays [25, 26], immunofluorometric assays [27] and multiplex proteomic assays [28, 29] generally reported higher detection limits and/or greater analytical variability.

**Table 1:** Characteristics of commonly used assay platforms to measure IGFBP7

Assay	First author, year	Limit of detection (ng/mL)	Total imprecisions	
			Intra-assay CV (%)	Inter-assay CV (%)
Electrochemiluminescence immunoassay(Elecsys®, Roche Diagnostics)	Ferreira[17], 2024	0.01	2%	5%
	Januzzi Jr [30], 2021	0.01	2%	5%
	Abou Kamar[15], 2024	0.01	2%	5%
	Gandhi[31], 2016	0.01	≤ 2%	≤ 5%
	Gandhi[32], 2017	0.01	≤ 2%	≤ 5%
	Tan[33], 2023	0.01	2.9%	< 6%
	Bracun [16], 2022	0.01	2%	5%
	Blum[34], 2021	0.01	2%	5%
	Kalayci[35], 2020	0.01	2%	5%
ELISA immunoassay(Roche Diagnostics)	Motiwala[36], 2014	0.10	3.5%	4.8%
	Barroso [37], 2016	0.10	3.5%	4.8%
	Gandhi[36], 2014	0.10	3.5%	4.8%
ELISA kit 7 (USCN Life Science Inc.)	Szyszkowska[25], 2024	0.2	< 10%	< 12%
Immunofluorometric assay(from R&D Systems, DY1334)	Hage[27], 2018	0.04	≤ 5%	≤ 8%
Standard immunosorbent assay (Abcam #ab213790)	Puar[26], 2023	0.63	7.2%	7.8%
IGFBP-7 Duoset Immunoassay (R&D Systems)	Adamson[38], 2023	0.04	4.7%	14.9%
Multiplex immunoassay(Proseek Multiplex96×96Olink Bioscience)	Sanders-van Wijk[28], 2020	0.03	9%	13%
The Cardiovascular Panel III (Olink Proteomics AB)	Brankovic[29], 2018	0.04	9%	13%
SomaScan Assay, v4.0(SomaLogic)	Shah[39], 2024	0.0042	N/A	N/A

*CV* coefficient of variation, *N/A* not available

### IGFBP7 and Heart Failure Development

In a discovery study, Shah et al. combined data from three extensively phenotyped, community-based cohorts (mean age 60–75 years): the Atherosclerosis Risk in Communities (ARIC), the Trøndelag Health Study (HUNT), and the Multi-Ethnic Study of Atherosclerosis (MESA), totalling over 24,000 participants, all free of HF at baseline [39]. IGFBP7 was measured using the SOMAscan v4 platform, a high-throughput, aptamer-based proteomic assay that quantifies over 4000 proteins from plasma samples. IGFBP7 was one of the 37 proteins consistently associated with incident HF across all cohorts, independent of traditional risk factors and natriuretic peptides, and was among the subset of proteins cross-sectionally linked to left ventricular (LV) and left atrial size and diastolic function. Mendelian randomization (MR) analyses further supported a potential causal role for IGFBP7, with genetically higher circulating levels associated with increased LV end-systolic volume and reduced LV ejection fraction. Of the 10 causal proteins identified in MR analyses [39], eight proteins, including IGFBP7, were annotated as druggable targets [40].

**Fig. 2:**
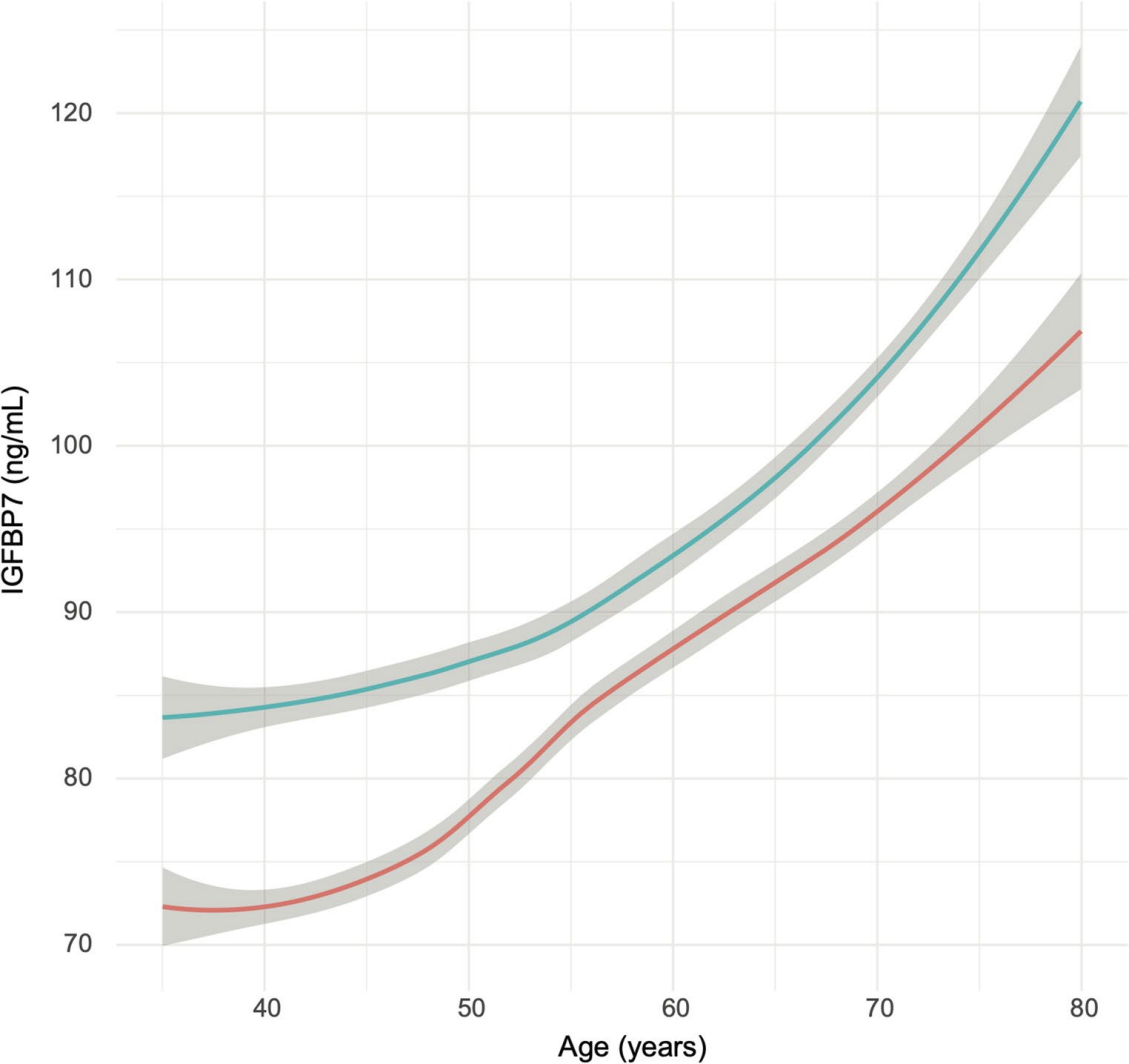
Median levels of insulin-like growth factor-binding protein-7 (IGFBP7) in men (in blue) and women (in red) with their corresponding prediction intervals (in grey)

In the Prevention of Renal and Vascular Endstage Disease (PREVEND) community-based cohort (mean age 53 years; 51% women), IGFBP7 levels were measured in 6125 individuals using the Elecsys^®^ electrochemiluminescence immunoassay (Roche Diagnostics), and showed a progressive increase with age in both sexes **(**Fig. 2**)** [15]. IGFBP7 was inversely associated with relative fat mass [15, 41], and strongly associated with renal dysfunction [15].

Over a median follow-up of 8.4 years, higher baseline IGFBP7 levels were strongly associated with incident HF in the overall population (HR 1.42, 95% CI 1.25–1.62), and this association remained significant after multivariable adjustment (HR 1.22, 95% CI 1.03–1.46). In 3879 individuals with repeated measurements approximately four years apart, temporal increases in IGFBP7 were independently associated with incident HF (HR 1.39, 95% CI 1.06–1.81).

## IGFBP7 and Echocardiographic Markers of Cardiac Stress

Table 2 summarizes key echocardiographic correlates of IGFBP7 across HF phenotypes. In a pioneering study of HF patients with reduced ejection fraction (HFrEF: *n* = 124) [36], IGFBP7 levels correlated with diastolic dysfunction, including increased left atrial volume index (LAVI), elevated transmitral E/A ratio, higher E/E′ ratio, and increased right ventricular systolic pressure (RVSP) - but not with LV size or systolic function. Longitudinally, cumulative exposure to elevated IGFBP7 was associated with progressive increases in LA size and RVSP.

**Table 2:** IGFBP7 and Echocardiographic Markers of Cardiac Stress

Authors (year)	HF subtype (population size)	Age range	Echo parameters related to higher IGFBP7
Gandhi [36] (2014)	HFrEF (*n* = 124)	63.4 years	• **↑** LAVi, Transmitral E/A ratio, E/e’ ratio and RVSP
Gandhi [31] (2016)	HFpEF (*n* = 160)	67-69 years	• **↑** E velocity, LAVi, estimated RVSP and transmitral E/A ratio
Barroso [37] (2016)	LVDD & HFpEF (*n* = 300)	54-73 years	• **↑** LAVi and E/e’ ratio
Hage [27] (2018)	HFpEF (*n* = 86) HFrEF (*n* = 79)	64-73 years	• **↑** E/e’ and E/A ratio – only in HFpEF patients
Januzzi [42], 2018	HFpEF (*n* = 228)	67–75 years	• **↑** Left atrial width, LAVi, E/e’ ratio, E/A ratio
Sanders-van Wijk [28] (2020)	HFpEF (*n* = 228)	73-78 years	• **↑** Mitral E velocity, E/e’ ratio, E/A ratio, and TR velocity
Kalayci [35] (2020)	Dyspnoeic patients with and without acute HF (*n* = 271)	59-67 years	• **↑** LAVi, RV diastolic and systolic area, and tissue Doppler E/e’ ratio • ↓ RV fractional area change • **↑** LV mass index, LVEDVi, LVESVi • ↓ LVEF
Tan [33] (2023)	HFpEF & HFrEF (*n* = 863)	63–72 years	• **↑** LAVi, E/A ratio, E/e’ ratio, peak TR velocity and IVC dimension • ↓ LVEDV and LVESV

In HF patients with preserved ejection fraction (HFpEF, *n* = 160; RELAX trial) [31], circulating IGFBP7 levels correlated with elevated left ventricular filling pressures, including transmitral E velocity, E/E′ ratio, LAVI, and estimated RVSP, with weaker correlations for the E/A ratio. Longitudinal increases in IGFBP7 over 24 weeks reflected worsening diastolic function. Baseline IGFBP7 was also inversely correlated with peak oxygen consumption (VO₂max) [13.2 (95% CI 11.1–16.1) vs. 11.1 (95% CI 9.9–13.4) mL/kg/min, *p* < 0.001], and rising IGFBP7 levels were modestly associated with declines in VO₂max over time. In another HFpEF cohort (*n* = 300) [37], serum IGFBP7 levels increased progressively from healthy controls, to individuals with asymptomatic left ventricular diastolic dysfunction (LVDD), and reaching the highest levels in overt HFpEF.

Proteomic analyses in a HFpEF cohort (*n* = 228, PROMIS-HFpEF) [28] identified IGFBP7 as a mediator linking comorbidity burden to cardiac remodelling and diastolic dysfunction. Together with other senescence- and inflammation-related markers such as TNF-R1 (tumour necrosis factor receptor 1), uPAR (urokinase plasminogen activator receptor), and GDF-15 (growth differentiation factor-15), IGFBP7 mediated associations between multimorbidity and diastolic and right-sided hemodynamic stress, specifically mitral E velocity, E/e′ ratio, and tricuspid regurgitation velocity.

Comparative studies across HF phenotypes show variability in IGFBP7 concentrations, with one study reporting higher levels in HFrEF [27] and another in HFpEF [18]. In these cohorts, IGFBP7 was associated with echocardiographic markers of diastolic dysfunction (E/e′ and E/A ratio) in HFpEF [18, 27], and with age and atrial fibrillation in HFrEF [27]. Additionally, in a mixed cohort of patients with and without acute HF (*n* = 271; ICON-RELOADED) higher IGFBP7 levels were not only correlated with diastolic dysfunction (LAVi, E/e′ ratio), but also with reduced LVEF [35].

## IGFBP7 and Heart Failure Progression

The prognostic value of IGFBP7 in HF was first described by Motiwala et al. in 142 patients with HFrEF [43]. Using ROC analysis, an IGFBP7 threshold of 117.8 ng/mL predicted adverse cardiovascular events at one year **(**Table 3**)**, with patients above this cut-point experiencing a markedly higher event rate than those consistently below it (64.3% vs. 26.0%, *p* = 0.005). Importantly, even partial reductions in IGFBP7 over time were linked to lower event rates, highlighting the additional prognostic value of serial IGFBP7 measurements. Similar findings were reported in a predominantly HFrEF cohort (*n* = 263), where both baseline IGFBP7 levels and their temporal changes (measured using Olink platform) independently predicted adverse outcomes after adjusting for clinical risk factors, NT-proBNP and hs-TnT **(**Table 3**)** [29]. These results were further corroborated in a larger HFrEF cohort from the DAPA-HF trial (*n* = 3158), in which IGFBP7 predicted cardiovascular death and worsening HF events after multivariable adjustment (including NT-proBNP and hs-TnT) [38]. Compared with patients in the lowest IGFBP7 tertile, those in the highest tertile had a 49% greater risk of the composite primary endpoint (HR: 1.49, 95% CI 1.17–1.89), as well as increased risk for individual components. Notably, increases in IGFBP7 of > 30 ng/mL over 12 months were associated with higher risk, whereas declines of > 30 ng/mL corresponded with lower risk, reinforcing IGFBP7 as a dynamic prognostic marker in HF.

**Table 3:** Characteristics of studies investigating associations of IGFBP7 with adverse clinical outcomes in heart failure patients

First author, year (reference)	Type of HF (study population size)	Age	Median circulating IGFBP7 levels (IQR) in ng/mL	Adverse Clinical Outcomes	Key Results
Motiwala [43], 2014	HFrEF (*n* = 142)	62–65 years	102.9 (83.8-140.9)	Total CV events at one year; Composite of worsening HF, hospitalization for HF, clinically significant ventricular arrhythmia, ACS, cerebral ischemia and cardiac death	OR = 0.83 per 10% time in response increase (95% CI 0.73–0.95) IGFBP7 > 117.8 ng/mL: 62.5% CV event rate IGFBP7 ≤ 117.8 ng/mL: 27.1% CV event rate IGFBP7 is associated with endpoints and lower IGFBP7 predicts fewer events.
Gandhi [32], 2017	HFpEF (*n* = 302)	73–74 years	218 (186–261)	All-cause mortality or CV hospitalization and composite (primary outcome)	aHR = 1.003 (0.998–1.007) Higher levels of baseline IGFBP7 had no significant association with outcomes after multivariate adjustment. Changes in IGFBP7 over 6 months showed no significant association with outcomes.
Hage [27], 2018	HFpEF & HFrEF (*n* = 165)	64–73 years	HFpEF: 102 (85–128) HFrEF: 152 (120–206)	HFpEF: All-cause mortality or HF hospitalization HFrEF: all-cause mortality, LVAD or HTx	HFpEF: aHR = 4.19 (95% CI 1.01–17.35) HFrEF: aHR = 0.72 (95% CI 0.24–2.14) IGFBP7 is associated with HF severity in patients with HFrEF, but not with HF prognosis. In HFpEF, it is associated with the composite outcome (markers and severity of HF, diastolic dysfunction and prognosis).
Brankovic [29], 2018	Predominantly HFrEF (*n* = 263)	67 years	N/A	Composite of HF hospitalization, cardiac death, LVAD and HTx	aHR = 2.72 (95% CI 2.06–3.60) Serially measured IGFBP7 levels correlate independently with the composite endpoint.
Bracun [16], 2022	HFpEF & HF(m)rEF (*n* = 2250)	74.2–83.7 years	T1: 99.4 (89.9-107.2) T2: 133.2 (124.7-143.7) T3: 195.1 (170.2-235.7)	Hospitalization or all-cause mortality or combined endpoint	Hospitalization: aHR = 1.75 (95% CI 1.25–2.46) ACM: aHR = 1.71 (95% CI 1.39–2.11) Combined: aHR = 1.44 (95% CI 1.23–1.70) IGFBP7 is an independent and robust prognostic biomarker for the endpoints for HFpEF and HF(m)rEF combined.
Tan [33], 2023	HFpEF & HFrEF (*n* = 863)	63–72 years	121 (99–156)	Composite of HF hospitalization and all-cause mortality	aHR = 2.01 (1.45–2.79) IGFBP7 is independently associated with endpoint and has a strong prognostic performance. There is no significant difference between HFpEF and HFrEF for the association between IGFBP7 and the primary outcome.
Adamson [38], 2023	HFrEF (*n* = 3158)	64.9–68.9 years	T1: 147 (43–169) T2: 193 (170–224) T3: 272 (225–864) Total cohort: 192 (158–246)	Composite of worsening HF event or CV event	T1: aHR = 1.00 (reference) T2: aHR = 0.94 (95% CI 0.74–1.20) T3 aHR = 1.49 (95% CI 1.17–1.89) Highest tertile of IGFBP7 is associated with the endpoint.
Szyszkowska [25], 2024	Predominantly HFpEF (*n* = 143)	72.2 years	HFpEF: 2.59 ± 1.85 HFmrEF: 2.75 ± 2.05	Duration of hospitalization, risk of rehospitalization, or mortality	Mortality: OR = 1.26 (95% CI 0.92–1.74) Exacerbation of HF symptoms AUC: 0.50 (95% CI 0.38–0.62) IGFBP7 is not significantly associated with endpoints.
Ferreira [17], 2024	HFpEF & HFrEF (*n* = 1125)	67.1–71.7 years	N/A	CVD or first hospitalization for HF	< T1: reference ≥T1 and < T2: aHR = 1.03 (95% CI 0.66–1.63) ≥T2: aHR = 2.00 (95% CI 1.28–3.10) Highest tertile of IGFBP7 is strongly related to the endpoint for both HFpEF and HFrEF combined.

*ACS *acute coronary syndrome, *AUC *area under the curve, *aHR *adjusted hazard ratio, *CI *confidence interval, *CV *cardiovascular, *CVD *cardiovascular death, *EF *ejection fraction, *HF *heart failure, *HF(m)rEF *heart failure with (mildly) reduced ejection fraction, *HFpEF *heart failure with preserved ejection fraction, *HTx *heart transplantation, *IGFBP7 *insulin-like growth factor-binding protein-7, *LVAD *left ventricular assist device, *LVSD *left ventricular systolic dysfunction, *N/A *not available, *OR *odds ratio, *T* tertile

Early small-scale studies in HFpEF yielded less consistent findings. Gandhi et al. reported that baseline IGFBP7 was associated with an increased risk of cardiovascular hospitalization or all-cause mortality in the I-PRESERVE trial (*n* = 302, median follow-up 3.6 years) [32], but this association was no longer significant after multivariable adjustment; changes in IGFBP7 levels over 6 months were also not associated with outcomes. Similarly, a study including 143 predominantly HFpEF patients [25] reported that although IGFBP7 correlated with NT-proBNP and echocardiographic markers, it was not associated with adverse clinical outcomes over two years of follow-up. By contrast, another study in chronic HF patients (*n* = 165) [27] found that elevated IGFBP7 was associated with adverse clinical outcomes in HFpEF (HR: 4.19, 95% CI 1.01–17.35) but not in HFrEF (HR: 0.72, 95% CI 0.24–2.14).

Larger, phenotype-inclusive cohorts have more consistently demonstrated the prognostic value of IGFBP7 across the HF spectrum. In the BIOSTAT-CHF study (*n* = 2250) [16], elevated IGFBP7 independently predicted HF hospitalization, all-cause mortality, and composite outcomes across LVEF categories during a median follow-up of 21 months. Subgroup analyses confirmed its prognostic value in both HFpEF and HFrEF; for each doubling of IGFBP7 concentration, the HR for HF hospitalization or mortality was 2.41 (95% CI 1.69–3.46) in HFpEF and 1.27 (95% CI 1.04–1.55) in HFrEF. Tan et al. followed 863 patients with chronic HF (36% HFpEF) for 4.6 years [33] and showed that IGFBP7 ≥ 110 ng/mL was independently associated with a 32% increased risk of HF hospitalization or all-cause mortality, with no significant interaction by LVEF; median plasma IGFBP7 levels were also comparable between HFpEF and HFrEF (126 versus 120 ng/mL). Ferreira et al. further confirmed these findings in 1125 HF patients from the EMPEROR-Reduced and EMPEROR-Preserved trials [17]. Participants in the highest IGFBP7 tertile had a two-fold increased risk of HF hospitalization or cardiovascular death (HR: 2.00, 95% CI 1.28–3.10), and a more than four-fold increased risk of renal events (HR: 4.66, 95% CI 1.61–13.53), independent of NT-proBNP, hsTnT, and GDF-15; additionally, 12-week changes in IGFBP7 concentrations were strongly associated with outcomes. No significant interaction by LVEF was reported.

## IGFBP7, Atrial Fibrillation, and Heart Failure

Atrial fibrillation (AF) commonly coexists with chronic HF and their combination leads to worse clinical outcomes than either condition alone [44]. Studies examining IGFBP7 in the context of AF have been limited; however, emerging data indicate that circulating IGFBP7 not only reflects AF risk in the general population but also strongly predicts HF risk in patients with AF. In a population-based cohort of 5884 adults (mean age 54 years, 52% female), higher IGFBP7 levels were independently associated with incident AF over a median follow-up of 6.4 years (HR: 1.29, 95% CI 1.07–1.52) [45]. Among 3691 patients with established AF (mean age 69 years, 28% female), those in the highest IGFBP7 quartile had a markedly increased risk of HF hospitalization (HR: 4.37, 95% CI 2.72–7.04); notably, elevated IGFBP7 also predicted HF hospitalization in 2812 AF patients without prior HF (HR: 3.48, 95% CI 1.94–6.24) [34].

## IGFBP7 Inhibition in Heart Failure

In a seminal study, Zhang et al. [18], identified cardiomyocytes as a primary source of circulating IGFBP7 in HF through a series of complementary experiments: (1) in patients with chronic HF, *IGFBP7* expression in myocardial tissue and IGFBP7 levels in plasma were markedly elevated compared to non-HF controls; (2) circulating IGFBP7 strongly correlated with myocardial levels (*r* = 0.70), supporting the heart as a principal source; (3) immunoblotting and immunofluorescence of cardiac biopsies localized IGFBP7 increase (in myocardial tissue) predominantly to cardiomyocytes; (4) in a murine model of pressure overload-HF, transverse aortic constriction (TAC) induced robust upregulation of *Igfbp7* mRNA and protein in cardiomyocytes; (5) in cultured cardiomyocytes, hypertrophic stimuli such as angiotensin II and phenylephrine induced *IGFBP7* expression, accompanied by redistribution of IGFBP7 protein to the cell membrane and secretory vesicles, consistent with active secretion under stress. Notably, in this study, *IGFBP7* upregulation was absent in microvascular endothelial cells, indicating cardiomyocyte specificity.

Next, they examined whether IGFBP7 influences cardiac remodelling via IGF-1/insulin signaling using wild-type and IGFBP7-deficient (*Igfbp7*^*⁻/⁻*^) mice under basal (non-stressed) conditions. They reported that in *Igfbp7*^*⁻/⁻*^ mice, IGF-1 protein levels in both myocardial tissue and plasma were reduced, consistent with IGFBP7 acting as a stabilizer or activator of IGF-1 signaling – a pathway that, although essential during development, has been implicated in driving cardiovascular senescence in aged myocardium [11]. They further explored the therapeutic potential of IGFBP7 inhibition in a murine model of TAC-induced HF using both genetic deletion and pharmacological approaches. *Igfbp7*^*⁻/⁻*^ mice displayed attenuated LV systolic dysfunction, reduced myocardial hypertrophy and fibrosis, and lower cardiomyocyte senescence compared with wild-type controls. Neutralizing monoclonal antibody therapy targeting IGFBP7 recapitulated these protective effects, confirming the potential for pharmacologic intervention. Mechanistically, IGFBP7 inhibition disrupted IGF-1 receptor signaling via the IRS/Akt pathway, increased nuclear localization of (transcription factor) FOXO3a, and promoted expression of anti-senescent and cytoprotective genes involved in oxidative stress resistance and DNA repair **(**Table 4**)** [46].

**Table 4:** Experimental studies targeting IGFBP7 in mice with heart failure

Cellular source of IGFBP7	Effect of IGFBP7 on IGF-1/Insulin axis	IGFBP7: pathophysiological mechanism	Effect of targeting IGFBP7
Cardiomyocytes [18]	• Focus is on IGFBP7 capacity to bind IGF1 receptor, thereby stimulating pro-aging signalling of IGF-1 in cardiomyocytes	• Autocrine action • Cardiomyocytes secrete IGFBP7 and this induces senescence in cardiomyocytes • IGFBP7 ➔ stimulation of IGF-1R/IRS/AKT-dependent suppression of FOXO3a ➔ prevents DNA repair and ROS detoxification ➔ accelerates HF progression	• Attenuated cardiac dysfunction by reducing cardiac inflammatory injury, tissue fibrosis and cellular senescence. • Attenuated pressure-overload-induced HF in mice.
Endothelial cells [19]	• Focus is on IGFBP7 capacity to bind to the insulin receptor, thereby blocking its metabolic role in cardiomyocytes	• Paracrine action • Replication stress due to pressure overload leads to upregulation of IGFBP7 expression in senescent endothelial cells ➔ subsequent secretion of IGFBP7 ➔ attenuation of oxidative phosphorylation in cardiomyocytes + inflammation ➔ HF progression	• Increased oxidative phosphorylation in cardiomyocytes and ameliorated cardiac dysfunction. • Attenuated pressure-overload-induced HF in mice.

*IGFBP7* insulin-like growth factor-binding protein, *IGF-1* insulin-like growth factor 1

In a subsequent TAC-induced HF study, Katoh et al. [19] focused on IGFBP7 derived from senescent endothelial cells (ECs). They reported that pressure overload triggered DNA damage (due to replication stress) and induced senescence in ECs. Single-cell RNA sequencing of murine and human failing hearts confirmed that IGFBP7 was predominantly expressed in senescent ECs, while cardiomyocytes showed downregulation of insulin signaling and oxidative phosphorylation pathways, suggesting a paracrine effect of (EC-derived) IGFBP7 on cardiomyocyte metabolism. Additionally, using three complementary strategies, the authors showed that: (1) cardiac overexpression of IGFBP7 via AAV9 vectors exacerbated cardiac dysfunction; (2) EC-specific IGFBP7 knockout ameliorated TAC-induced dysfunction; and (3) vaccination using IGFBP7 peptide epitopes improved cardiac function (in TAC-induced HF) by restoring oxidative phosphorylation in cardiomyocytes.

Taken together, Zhang et al. show that IGFBP7, secreted from stressed cardiomyocytes, drives cardiomyocyte senescence via increased IGF-1R/IRS/Akt signaling, whereas Katoh et al. show that IGFBP7, secreted from senescent ECs, disrupts cardiomyocyte metabolic homeostasis through paracrine inhibition or downregulation of insulin signaling in cardiomyocytes **(**Table 4**)** [46]. Despite these mechanistic differences, both studies converge on the conclusion that IGFBP7 inhibition confers cardioprotection in experimental pressure-overload HF.

## Discussion

Circulating IGFBP7 reflects aspects of HF pathophysiology – particularly diastolic function and cardiovascular ageing – not fully captured by traditional biomarkers such as natriuretic peptides, and not directly targeted by current guideline-directed HF therapy. Evidence from smaller HFpEF studies suggests that therapies such as sildenafil and sacubitril/valsartan may modestly lower IGFBP7 levels. In the RELAX trial [31], 160 HFpEF patients receiving sildenafil over 24 weeks showed a slight decrease in circulating IGFBP7 (− 1.5 ng/mL) whereas levels increased in the placebo arm (+ 13.6 ng/mL; *p* = 0.001). Additionally, in a subset of 228 HFpEF patients from the PARAMOUNT trial [42], sacubitril/valsartan led to a small but statistically significant reduction in IGFBP7 compared with valsartan alone (adjusted treatment effect: −7%; *p* < 0.001). By contrast, studies evaluating SGLT2 inhibitors reported no meaningful effect on circulating IGFBP7. In DAPA-HF [38], 3158 HFrEF patients were assessed at baseline and 2493 at one year, IGFBP7 levels increased in both dapagliflozin and placebo arms, with no significant between-group difference (treatment ratio: 0.99, 95% CI 0.97–1.01). Similarly, in the EMPEROR programme [17], among 594 HFrEF and 531 HFpEF patients, no significant changes in IGFBP7 were observed with empagliflozin at week 12 (1.04, 95% CI 1.01–1.07) or week 52 (1.02, 95% CI 0.99–1.05). Collectively, these findings indicate that guideline-directed HF therapies do not consistently reduce IGFBP7 levels, highlighting the potential value of therapies that specifically target IGFBP7-mediated pathways.

Preclinical studies support the therapeutic potential of focused IGFBP7 inhibition; [18, 19] however, translation to humans may present certain challenges. Excessive clearance of senescent cells could interfere with tissue repair, as transient senescence contributes to matrix remodelling, angiogenesis, and wound healing; [47] this may be particularly relevant in the context of myocardial infarction, where reparative senescence facilitates scar formation and structural stabilization. Furthermore, senescence serves as a tumour-suppressive mechanism by preventing proliferation of damaged cells [48, 49], and studies show that IGFBP7 itself may function as a tumour suppressor in certain malignancies (e.g., breast, liver) and as pro-tumorigenic in others (e.g., colorectal, gastric) [50]. Systemic IGFBP7 blockade could therefore entail divergent risks depending on cardiovascular status, tissue context, and cancer susceptibility.

## Future directions

Translating IGFBP7-targeted strategies from preclinical studies to clinical HF therapy will require a systematic, multifaceted approach **(**Table 5**)**. First, the context-dependent biology of IGFBP7 - shaped by cell type, senescence state, and receptor interactions – needs clarification. Defining the dominant cellular sources and targets of IGFBP7, and reconciling mechanistic differences between cardiomyocyte- and endothelial-derived IGFBP7, is essential for rational therapeutic development. Second, preclinical studies should expand beyond pressure-overload models to ischemic models, such as myocardial infarction-induced HF, to evaluate the cardiovascular consequences of IGFBP7 inhibition and its impact on reparative senescence and scar formation. Third, given the context-dependent role of IGFBP7 in tumour biology, studies in cancer-prone models are necessary to evaluate whether IGFBP7 inhibition could accelerate tumour progression, particularly under cardiovascular stress. Fourth, human-relevant models - such as iPSC-derived cardiomyocytes and endothelial cells engineered into senescent and non-senescent states - can help dissect cell-specific signalling, resolve mechanistic discrepancies, and refine translational strategies. Finally, additional studies are needed to elucidate how guideline-directed HF therapies influence circulating IGFBP7, as this may inform the potential added value of IGFBP7-targeted strategies.

**Table 5:** Translational checklist for IGFBP7-targeted strategies

Research priority	Key focus	Purpose
Mechanistic resolution	Define dominant cellular sources of IGFBP7 (cardiomyocytes vs. endothelial cells) and reconcile conflicting mechanisms	To clarify context-dependent IGFBP7 biology and guide targeted therapeutic development
HF model expansion	Evaluate IGFBP7 inhibition in ischemic HF (e.g., myocardial infarction models)	To determine cardiovascular effects, including impact on reparative senescence and scar formation
Cancer risk evaluation	Test IGFBP7 inhibition in cancer-prone preclinical models of HF	To assess potential tumour-promoting or tumour-suppressive consequences under cardiovascular stress
Human-relevant models	Use iPSC-derived cardiomyocytes and endothelial cells (senescent vs. non-senescent)	To dissect cell-specific signalling, resolve mechanistic discrepancies, and strengthen translational relevance
Interaction with GDMT	Investigate whether (and how) guideline-directed HF therapies influence circulating IGFBP7 levels	To determine whether IGFBP7-targeted therapies would provide additional benefit beyond standard HF treatment

## Concluding Remarks

IGFBP7 is a senescence-associated biomarker reflecting diastolic dysfunction and cardiovascular ageing in HF. Preclinical studies indicate that IGFBP7 inhibition attenuates adverse cardiac remodelling and slows HF progression, positioning it as a promising candidate for future therapeutic development. Ongoing research into IGFBP7-targeted strategies can help expand HF treatment beyond conventional hemodynamic and neurohormonal approaches by directly addressing the biology of cardiovascular ageing.

## Data Availability

No datasets were generated or analysed during the current study.
